# Stimuli-Responsive Microgels and Microgel-Based Systems: Advances in the Exploitation of Microgel Colloidal Properties and Their Interfacial Activity

**DOI:** 10.3390/polym10040418

**Published:** 2018-04-09

**Authors:** Garima Agrawal, Rahul Agrawal

**Affiliations:** 1 Department of Polymer and Process Engineering, Indian Institute of Technology Roorkee, Saharanpur Campus, Paper Mill Road, Saharanpur 247001, Uttar Pradesh, India; 2 Laboratory of Pathology, Center for Cancer Research, National Cancer Institute, National Institutes of Health, Bethesda, MD 20892-1500, USA; rahulagrawal10487@gmail.com

**Keywords:** microgels, stimuli-responsive, interface, catalysis, tissue engineering

## Abstract

In this paper, recent developments in the chemical design of functional microgels are summarized. A wide range of available synthetic methods allows the incorporation of various reactive groups, charges, or biological markers inside the microgel network, thus controlling the deformation and swelling degree of the resulting smart microgels. These microgels can respond to various stimuli, such as temperature, pH, light, electric field, etc. and can show unique deformation behavior at the interface. Due to their switchability and interfacial properties, these smart microgels are being extensively explored for various applications, such as antifouling coatings, cell encapsulation, catalysis, controlled drug delivery, and tissue engineering.

## 1. Introduction

Microgels are aqueous, crosslinked, ultrahigh molecular mass containing polymeric particles, which represent a special class of colloids due to various advantageous properties, including tunable architecture, high porosity, and adjustable dimensions [1,2,3,4]. The most interesting feature that puts microgels in the category of “smart materials” is their unique capability to adjust their dimensions in response to external stimuli, such as temperature, pH, ionic strength, and solvent quality [2,5,6,7,8,9]. Their excellent colloidal stability, ease of synthesis, and post-modification, large surface area enabling encapsulation of the desired cargo makes them potential candidates for antifouling coatings, drug and gene-delivery, tissue engineering, catalysis, water purification, cosmetic applications, and responsive macroscopic materials [10,11,12]. Several review articles can be found in the literature dealing with different fundamental aspects of microgels and the readers are referred to them for detailed information [13,14,15,16,17,18,19,20].

Microgels are soft with a fuzzy surface, having dangling chains when they are swollen, while they behave as hard colloidal particles in the shrunken state [21]. In the highly swollen state, microgels are comprised of an open structure with a diffuse outer boundary and well-solvated inner segments. This results in high mobility of solvent and solute molecules, and the network expands until the limit of elasticity of the chains is achieved. This open structure allows swelling/deswelling of microgels and network deformation in response to the surrounding environment [22]. Hence, in contrast to conventional hard particles, these microgels can exhibit unique interfacial behavior when located at fluid or solid interfaces without necessarily being amphiphilic. Further, the stability of the microgel-stabilized emulsions can be tuned by taking advantage of the stimuli-responsive characteristic of microgels [23]. Due to the tunable emulsion properties, these microgel-based emulsions are useful in biomedicine, catalysis, food products, and cosmetics [24,25].

Microgels with desired architecture and chemical versatility can be designed by the optimization of synthetic approach to ensure their successful use for targeted application [19]. These functional microgels exhibit faster response in comparison to their bulk counterparts, possess higher interfacial area per unit mass leading to greater exchange rates, and hence open a new pathway for biomedical applications [26,27].

In this paper, a brief overview will be given for the most important and relevant approaches of fabricating microgels with desired functionality, architectural versatility, and deformability. Further, the unique behavior of these microgels at the fluid or solid interface based on their ability to deform will be highlighted. Additionally, special attention will be given to recent advances in the application of microgels in the colloidal state, as well as at interfaces, including in antifouling coating, controlled release, and tissue engineering.

## 2. Chemical Design of Functional Microgels

The selection of a suitable synthetic strategy is crucial to fabricating microgels with controlled dimensions, polydispersity, and physical and chemical properties to achieve their successful use for desired applications. Synthetic approaches for the fabrication of functional microgels can be mainly categorized as: (a) precipitation polymerization; (b) miniemulsion polymerization; and (c) microfluidic method.

**Scheme 1:**
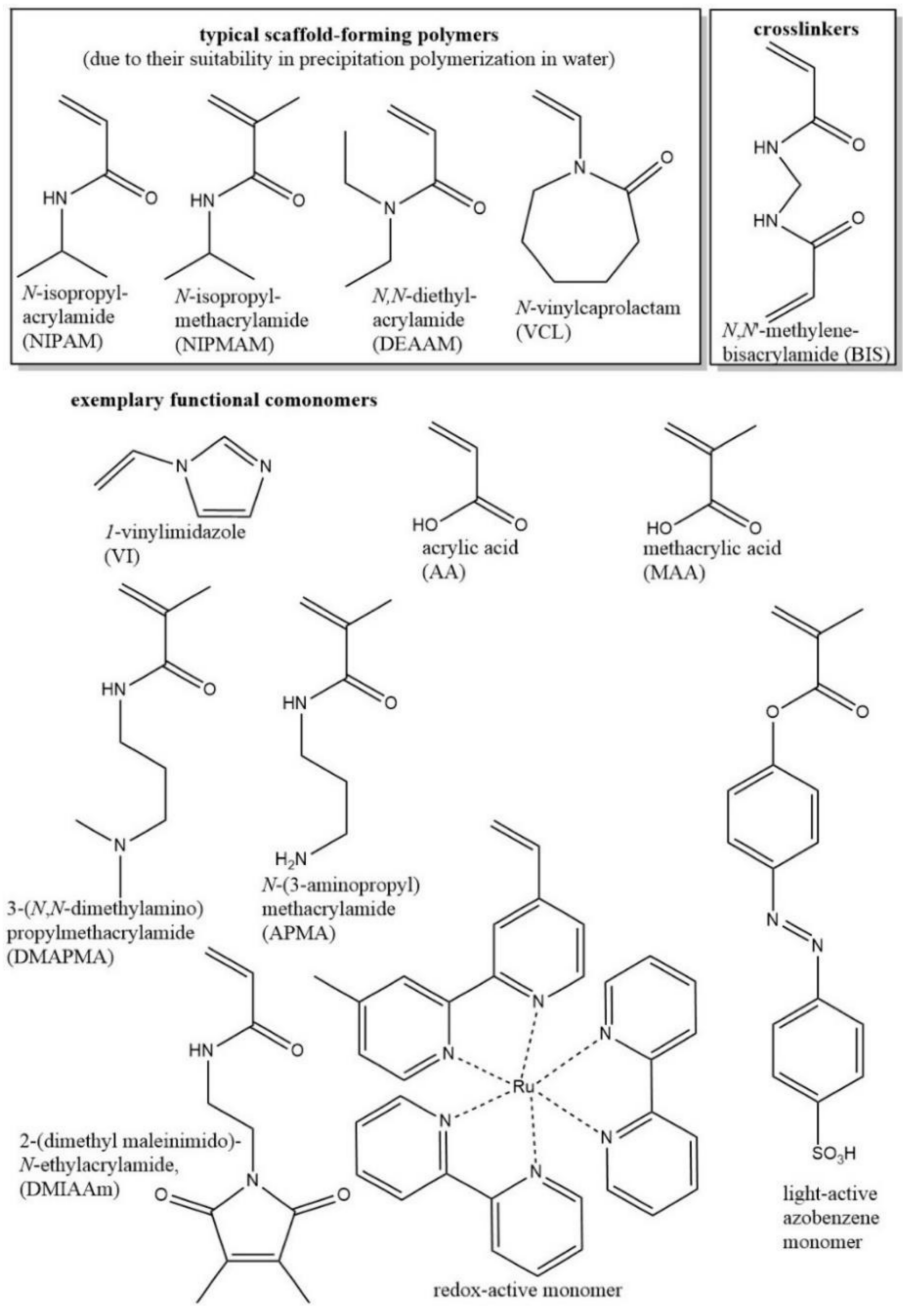
Chemical structures of various monomers used for designing functional microgels. (Reprinted with permission from [21]).

Precipitation polymerization is one of the most frequently used methods for the preparation of monodispersed microgel particles with tunable size [19,28]. Poly(*N*-isopropylacrylamide) (PNIPAM)-, poly(*N*-vinylcaprolactam) (PVCL)-, poly(*N*,*N*-diethylacrylamide) (PDEAAM)-, and poly(*N*-isopropylmethacrylamide) (PNIPMAM)-based responsive microgels have been synthesized frequently by using this method [29,30,31]. In this approach, all the ingredients including monomer, crosslinker, and initiator are dissolved in solvent, and the polymerization is started. Precursor particles are formed by collapse polymer chains as the reaction temperature is higher than the volume phase transition temperature (VPTT) of the monomer [29]. Newly formed polymer chains continue to add on these precursor particles, and the polymer chains are kept in position by using crosslinkers [32]. The size of these microgel particles can be adjusted by tuning the solvent composition, crosslinker content, addition of surfactant during synthesis, reaction temperature, initiator content, and monomer/comonomer ratio. A vast variety of functional microgels have been prepared so far by using various comonomers during synthesis, such as acrylic acid (AA) [20,33,34], methacrylic acid (MAA) [35], vinyl imidazole (VIm) [36,37,38], and aminoethyl methacrylate hydrochloride (AEMA) [29] (Scheme 1) [21]. Additionally, various microgels decorated with small active molecules [38], polymer chains [39,40], and proteins [41] have also been developed via post-modification for various applications. Hybrid microgels decorated with a variety of metal nanoparticles, such as Ag [42,43,44], Au [45,46,47,48], Pd [49], Pt [50,51], semiconductor particles [52], and metal oxides, such as Fe_3_O_4_ [53], Fe_2_O_3_ [54], ZnO [55], sulphides, and other inorganic materials, such as CdS [56], CuS [57], ZnS [58], CdTe [59], and CaCO_3_ [60], have also been reported in the literature for sensor, catalysis, and drug delivery applications.

The advantages of precipitation polymerization are: (a) the potential to carry out the polymerization in batch, semibatch, or continuous fashion; (b) microgels with controlled size (from 100 nm to 3 µm) and narrow particle size distribution are obtained; (c) different comonomers and nanoparticles can be integrated during the synthesis; and (d) microgels with different layers of shells having varied chemical composition can be prepared [19]. However, as the polymerization is carried out at higher temperatures, it is not suitable for the incorporation of temperature-sensitive biomolecules. Additionally, the formation of sol fraction occurs during the polymerization process, which needs to be removed later.

The water-in-oil (W/O) emulsion method involves polymerization of monomers and comonomers in water droplet stabilized by the surfactant in the presence of a crosslinker. In recent years, the miniemulsion process has been extensively explored as a versatile tool for designing microgels based on synthetics and biopolymers [61,62]. One main difference between the emulsion and miniemulsion methods is the size of the droplet. In the emulsion method, the droplets are formed by mechanical stirring, whereas in the minielmulsion method, kinetically stable small droplets ranging from 50 to 500 nm in size are generated by applying high shear stress [10]. These droplets lead to the formation of stable microgel particles by using crosslinking agents such as *N*,*N*′-methylene-bis-acrylamide (BIS) during polymerization [61]. This method helps to develop highly charged microgel particles, and the microgels based on hydrophilic monomers can be prepared via the inverse miniemulsion method [19,21]. However, this approach requires a large amount of surfactant during synthesis. Additionally, the particle size and polydispersity index are higher as compared with those of the microgels prepared by precipitation polymerization. A vast variety of microgel systems, such as polyacrylic acid (PAA), polyacrylamide (PAAM), PNIPAM, and poly(hydroxyethylmethacrylate) (PHEMA), decorated with different functionalities has been reported in the literature by using the miniemulsion process [61,62]. Furthermore, this process has been extended for designing complex microgel structures decorated with redox active units, light sensitive units, such as azobenzene and spyropyran derivatives, etc. [34,63,64,65,66,67,68,69]. We suggest several research articles, review articles, and book chapters for detailed information on micro/nanogels synthesis by miniemulsion polymerization [34,62,70,71,72,73,74,75,76].

A variety of microgels with sizes ranging from 1 to 30 μm have been developed by using the microfluidic technique [77]. In this approach, monodispersed droplets of monomers or prepolymers are formed by breaking up liquid streams, followed by physical or chemical crosslinking. The characteristic of this method is that it allows the formation of microgel particles with larger size (from 1 to 30 µm) and anisotropy [78,79]. Size and monodispersity of microgels can be controlled by adjusting the viscosities, polarities, and flow rates of the fluids. Dextran (Dex) microgels [80], microgels loaded with glucose oxidase (GOx) and horseradish peroxidase (HRP) [81], alginate microgels [82], cell-laden PEG maleimide microgels [83], and PNIPAM microgels [84] are some of the examples prepared by this approach. Further, this method has also been extended for the formation of janus and ternary particles [85,86].

The coacervation and desolvation method and the particle replication in non-wetting templates method (PRINT) have also been used in the literature for the fabrication of microgels with tunable properties. The readers are referred to various articles, which summarize the use of these methods [87,88,89,90,91,92,93,94].

## 3. Deformation of Microgels at Interfaces

Microgels are aqueous, crosslinked, soft polymeric porous particles swollen with a solvent, and they exhibit enhanced colloidal stability due to their macromolecular elasticity [20]. Microgels can deform and adapt their size and shape according to their surrounding environment. It is evident from the literature that microgels can translocate pores under physiological pressure during renal filtration, even though the pores are only one-tenth of the particle size [95]. On the other hand, it has been observed that when a colloidal crystal is formed using microgels with different sizes, the bigger particles fit into the lattice of smaller particles [96]. Therefore, to develop smart systems based on microgels, it is crucial to understand the interfacial behavior of these soft colloids and their dynamics at various interfaces. As the adsorption of such soft macromolecular particles enhances steric repulsion of the chains, it is also important to consider the surface-induced scission of covalent bonds based on the crosslinking gradient and the strength of adsorption [97,98]. Here, we provide the readers with a brief overview about the deformation behavior of microgels at air-solid, air-liquid, and liquid-liquid interfaces.

### 3.1. Microgels at Air-Solid Interfaces

**Figure 1:**
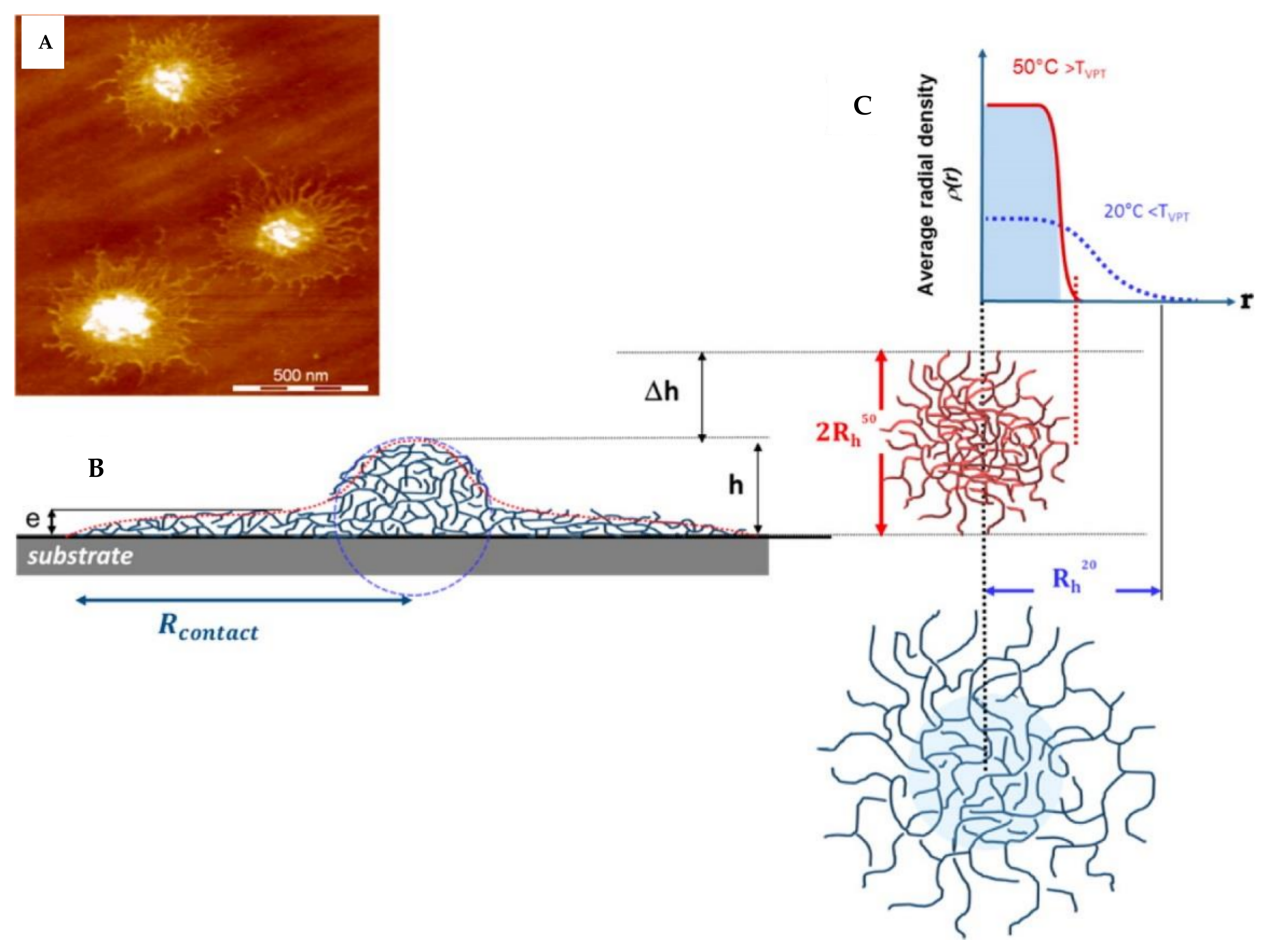
Height image of dry microgel on a solid surface (**A**); scheme of the height profile (**B**); density profile of a swollen and collapsed microgel (**C**) according to Ref. [105] to highlight the relation between the dense core and height, as well as the hydrodynamic radius and contact area. (Reprinted with permission from [22]).

When the microgel adsorbs from the bulk solution to a solid surface, the monitored macromolecular structure exhibits expansion parallel to the surface and compression perpendicular to the surface, showing substantial deformation resulting in a pancake-like structure (Figure 1) [22]. Here, height (*h*) represents the thick, dense core of the microgels, the contact radius (*R_cont_*) corresponds to the thin, extended outer layer of the microgel, and the hydrodynamic radius (*R_h_*_,20_) displays the most expanded conformation of the microgel in solution at a temperature of 20 °C, while *R_h_*_,50_ exhibits the collapsed state of the microgel in solution above the VPTT. It is reported that the spreading of the microgel at the interface is governed by the density of the network or conformational flexibility [99,100,101,102,103,104]. Based on the crosslinking degree of the polymer network, it is observed that the loosely, crosslinked microgels undergo large deformation due to higher flexibility of the polymer chains [59,60]. As the corona of the microgels is less crosslinked compared with the core structure, the spreading of polymer strands is more prominent at the periphery, and thus the outer shell undergoes more significant deformation compared with the core [22,105,106,107,108,109].

**Figure 2:**
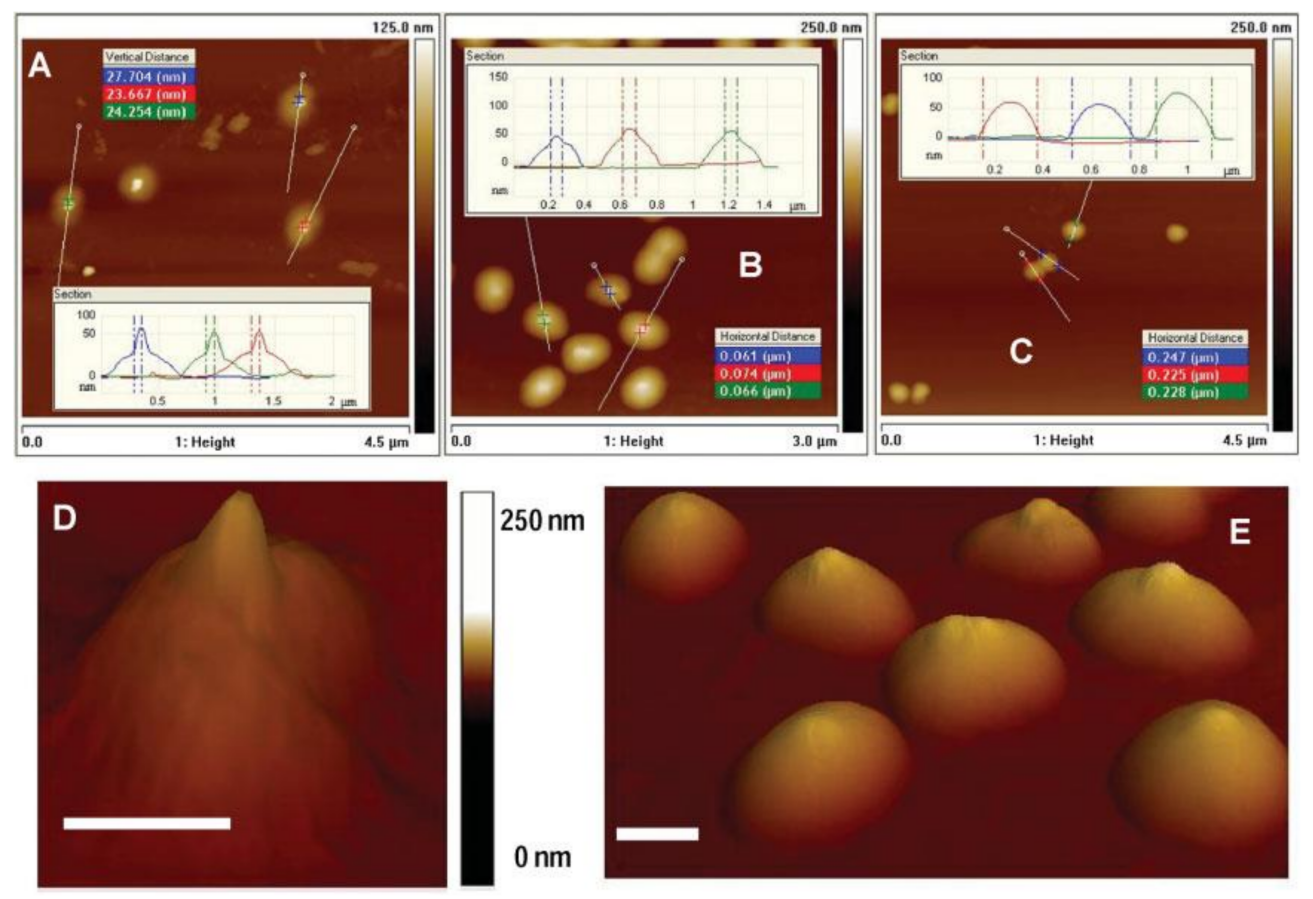
4.5 µm × 4.5 µm (**A**,**C**) and 3.0 µm × 3.0 µm (**B**) atomic force microscopy (AFM) topography images of Au@PNIPAM particles with 5% (**A**); 10% (**B**); and 17.5% (**C**) crosslinker. 3D plots of Au@pNIPAM composite particles with 5% (**D**) and 10% crosslinker (**E**). Scale bars represent 100 nm. (Reprinted with permission from [110]).

Contreras-Cáceres et al. studied the influence of bisacrylamide (BIS) crosslinker content on the deformation of Au@PNIPAM particles on a silicon surface using atomic force microscopy (Figure 2) [110]. Tapping mode atomic force microscopy (AFM) images of dried samples confirmed the core shell morphology. AFM results exhibited that the gold core protruded from the polymer shell in cases of 5% and 10% crosslinker content (Figure 2A,B) owing to the higher spreading of the shell. On the other hand, particles prepared with 17.5% crosslinker content did not show the gold core protrusions and thus confirmed the higher rigidity of the shell leading to less deformation (Figure 2C). 3D images of the samples prepared with 5% and 10% crosslinker content showed clear differences in the extent of the gold protrusion and the shell deformation (Figure 2D,E).

Schmidt et al. used a combination of AFM and ellipsometry to study the behavior of poly(NIPAM-*co*-acrylic acid) microgels in adsorbed state [111]. Microgels were prepared by using 2 mol % and 10 mol % BIS content and then they were spin coated on silicon wafers at pH 2. AFM measurements indicated dense packing along with strong flattening of the microgels on the substrate. Ellipsometry experiments showed that the average thickness of the films was 30 nm in the dried state and 400 nm in the swollen state. It was revealed that flattening behavior and reversible swelling/deswelling at the VPTT was more pronounced in the case of microgels prepared by using 2 mol % crosslinker.

Wellert et al. investigated poly(NIPAM-*co*-acrylic acid) microgels in solution and in the adsorbed state on a silicon surface coated with poly(allylamine hydrochloride) [112]. The temperature-dependent behavior of 5 mol % and 20 mol % acrylic acid containing microgels was analyzed using atomic force microscopy. The experimental results at 20 °C and 50 °C showed that the microgels were able to exhibit temperature-dependent deswelling even in the adsorbed state. This behavior was further confirmed by grazing incidence small angle neutron scattering (GISANS) experiments based on the change in the specular intensity. The comparison of results in the bulk and adsorbed state revealed that the solid substrate exhibits significant effect on temperature-dependent behavior and leads to the suppression of the divergence of internal fluctuations in the adsorbed microgels.

Taking advantage of microgel deformation, azetidinium-functionalized branched poly(ethylene imine) (PEI)-based antimicrobial ultrathin films have been developed by Chattopadhyay et al. using physically crosslinked microgels on mica and a highly oriented pyrolytic graphite surface [113].

Furthermore, electrostatic interaction can also take place with the surface. Microgels exhibit irreversible attachment to hydrophobic graphite surfaces, while limited adsorption is exhibited in the case of a platinum surface. High microgel coverage of the surface can be achieved if the microgels are adsorbed onto a surface in collapsed state, which in turn can be useful to develop thermo-switchable cell culture substrates and efficient switchable barriers for temperature-induced molecular transport through polymer multilayers [21].

### 3.2. Microgels at Air-Liquid Interfaces

**Figure 3:**
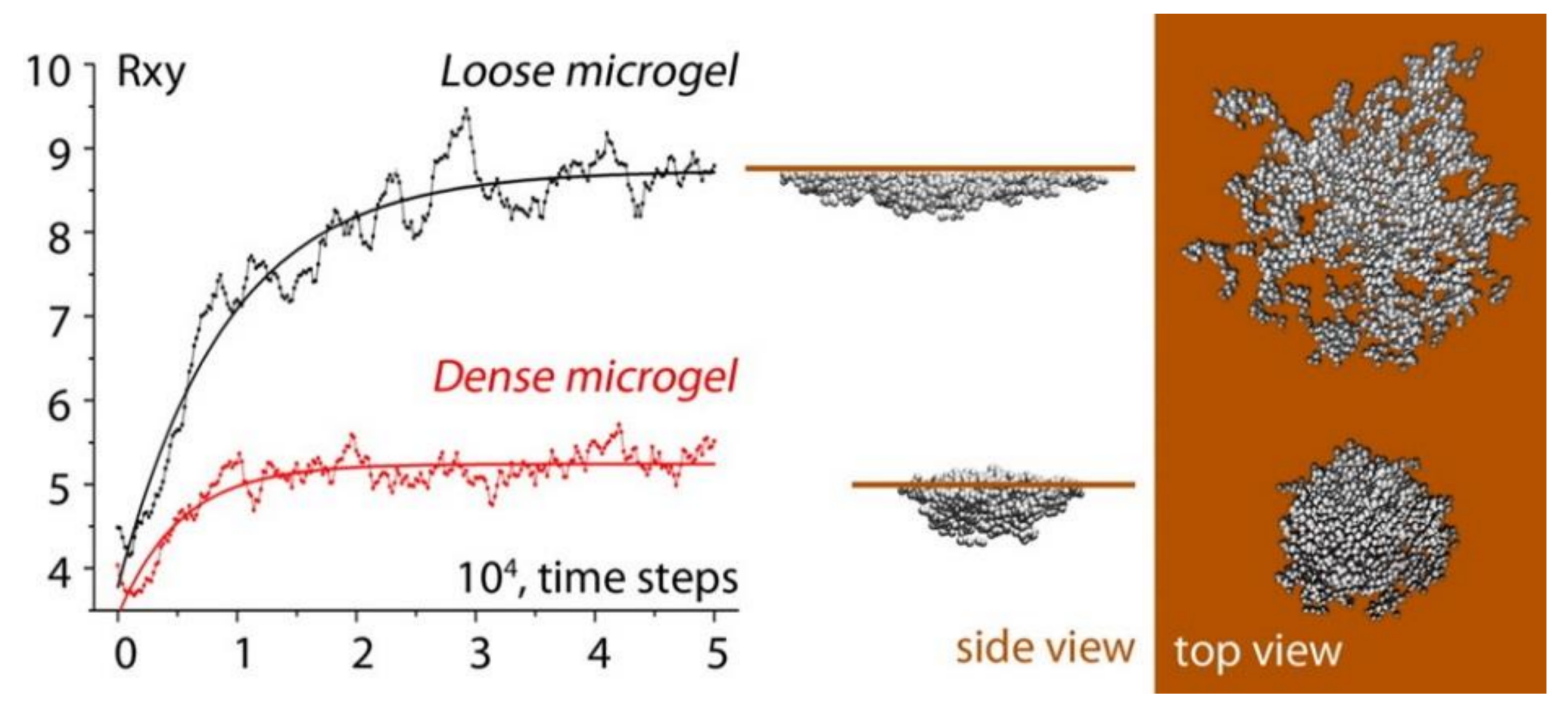
Average lateral diameter *R*_xy_ of the adsorbed dense and loose microgel particles as a function of time; steps and snapshots of the equilibrium structures. (Reprinted with permission from [22]).

The adsorption of microgels at the air/water interface lowers the interfacial tension by reducing the system free energy. The measurement of the surface tension (γ) as a function of time indicates that the surface tension decreases with the adsorption of microgels regardless of their crosslinking degree and reaches a steady-state equilibrium value of 46 mN/m in the case of 50 mol % vinyl caprolactam (VCL) microgel, while PNIPAM microgels lead to the equilibrium surface tension of ~42 mN/m [22,102]. Furthermore, the time required to reach the equilibrium is significantly affected by the crosslinking degree. It is observed that the microgels with low crosslinking density spread faster at the interface because of the lower elastic moduli and thus lead to fast reduction in the interfacial tension at the beginning. Later, the spreading rate slows down when the system moves towards the equilibrium state. Microgels with low crosslinking density show more deformation (*R*_xy_ ≈ 15.7 and height *R*_z_ ≈ 2.3) in comparison with highly crosslinked microgels (*R*_xy_ ≈ 11.1 and *R*_z_ ≈ 2.9) and hence a lower amount of low crosslinked microgels is required to cover the water/air interface, and saturation is achieved faster (Figure 3) [22].

### 3.3. Microgels at Liquid-Liquid Interfaces

Many polymers, such as polyethylene oxide, PNIPAM, etc., exhibit interface active properties and adsorb to the interface even without being amphiphilic [102,114,115]. Here, micelles are formed based on segregational self-assembly [21]. Microgels that do not show self-assembly in bulk can adsorb to the interface, and in this process, deformation is observed parallel to the interface. Microgel particles cover the maximum interface, and detrimental oil/water contacts are prevented.

**Figure 4:**
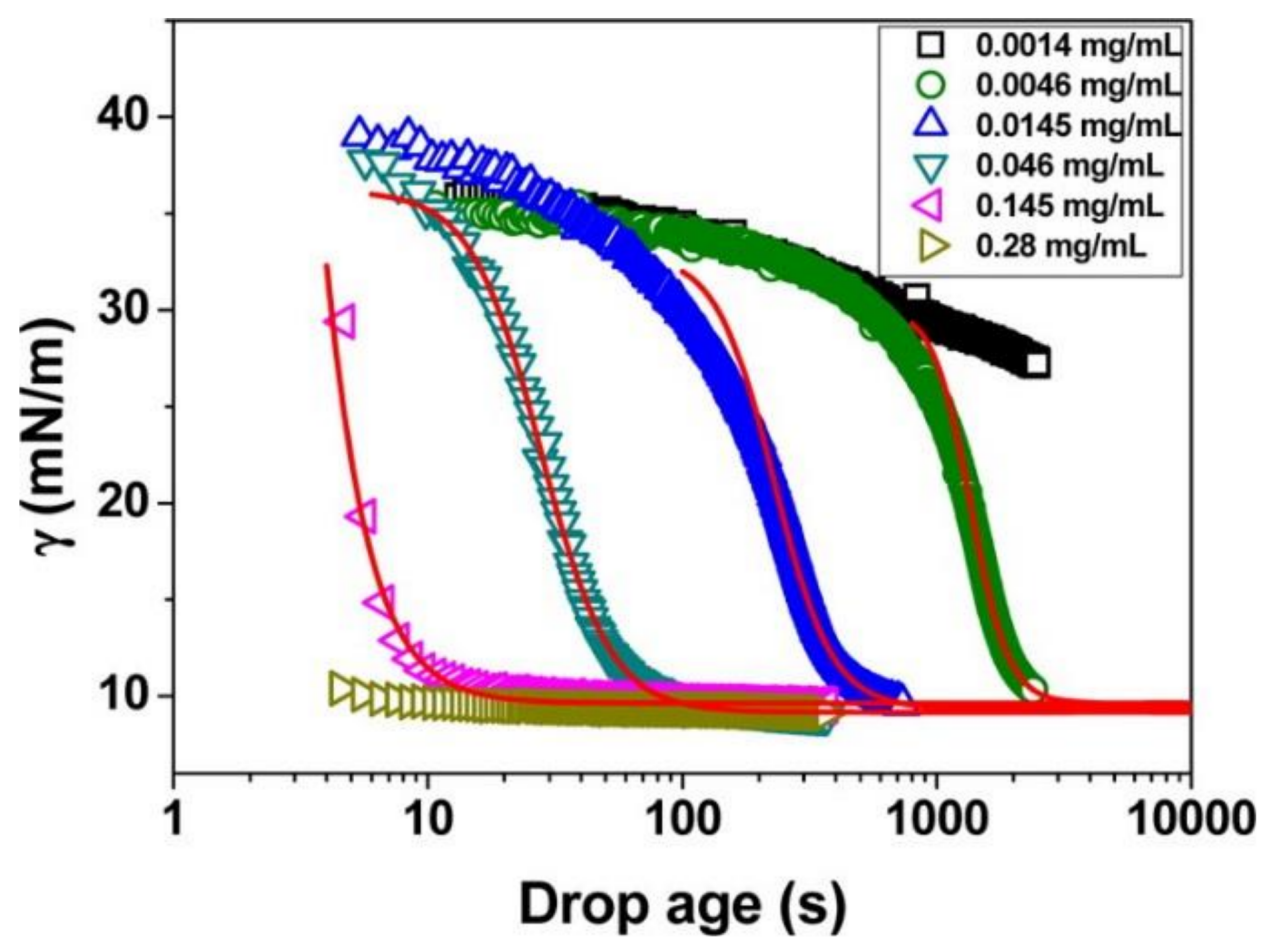
Time dependence of the toluene/water interfacial tension with PVCL/NIPMAm (5:1) microgels of various concentrations at 24 °C. (Reprinted with permission from [99]).

The interfacial tension of toluene against the aqueous solutions of PVCL/PNIPMAM microgels at different concentrations is displayed in Figure 4 as γ–log(time) plots [99]. The interfacial tension decreases as a function of time, and after certain time period, an equilibrium value is achieved. Additionally, the increase in the concentration of microgels leads to the reduction in the time required for reaching the equilibrium state. Similar to the dynamic interfacial behaviors of the polymer-grafted, inorganic nanoparticles and proteins, the γ–log(time) plots of the copolymer microgels exhibit three distinct regimes [116,117]. In the first stage, the interfacial tension decreases slightly (induction regime), followed by a sharp decline in the interfacial tension (second regime), and in the third regime, interfacial tension reaches a quasi-equilibrium value [99].

**Scheme 2:**
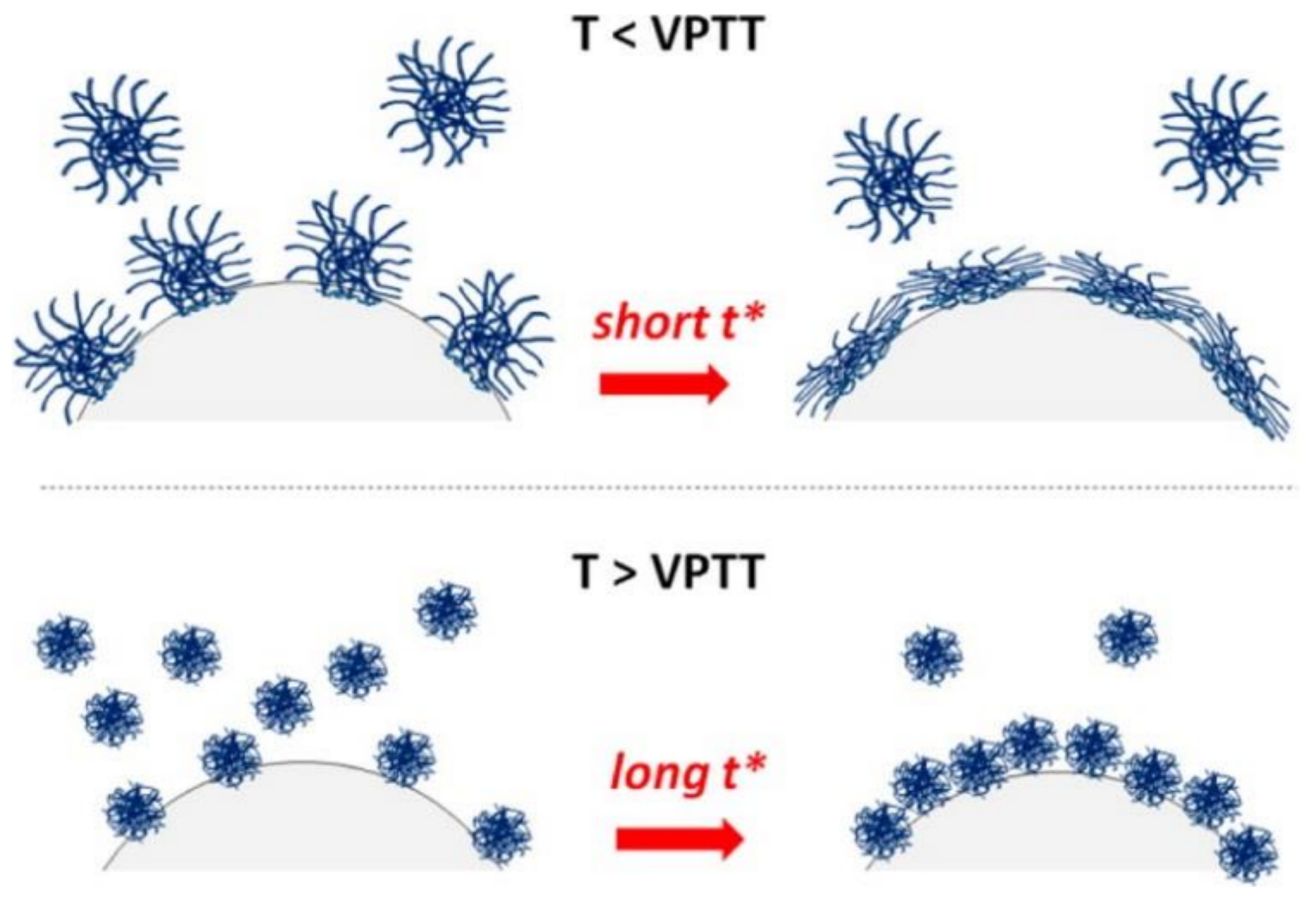
Adsorption of microgels below and above volume phase transition temperature (VPTT). t* is a semi-empirical parameter having the unit of time. (Reprinted with permission from [99]).

Furthermore, the interfacial behavior of microgels is also influenced by their volume phase transition temperature as displayed in Scheme 2 [99]. At *T* < VPTT, the microgel particles are swollen, and they spread at the interface covering it as much as possible. This may also lead to the formation of chain bridges between the adjacent microgel particles [101,103,118]. On the other hand, at *T* > VPTT, the microgel particles are shrunken, and hence a higher concentration of microgels is needed to cover the interface. Dense packing and jamming leads to lower mobility, and therefore, a strong increase of t* is observed at *T* > VPTT [119,120,121].

## 4. Smart Systems Based on Microgel-Stabilized Emulsions

The potential of microgels to stabilize the oil/water interface is primarily influenced by their adsorption capability at the interface. When microgel particles adsorb at the oil/water interface, the interfacial tension is reduced, leading to a microgel-stabilized emulsion [118,122,123,124,125,126]. This is a new type of emulsion stabilized by microgel particles and is also known as a “Mickering emulsion”, compared with the conventional “Pickering emulsion” where solid colloidal particles are used to stabilize the oil/water interface [118,127].

As the microgels are stimuli-sensitive and can respond to the external stimuli, such as temperature, pH, solvent composition, ionic strength, etc., the stability of the emulsion is highly influenced by the surrounding environment [101,103,128]. Hence, these Mickering emulsions can be broken once a stimulus is applied and thus contrast with the conventional Pickering emulsions, which lack the action of deformation. Monteux et al. reported the effect of temperature on the interfacial properties of temperature-responsive PNIPAM-based microgels adsorbed at the docosane/water interface [126]. It was observed that below the VPTT, the interfacial tension reduces with increasing temperature owing to the formation of a dense layer because of the decrease of the excluded volume interactions. On the other hand, the interfacial tension increases with temperatures above the VPTT caused by loosely packed microgels at the interface. The variation in temperature influences the hydrophilic/hydrophobic balance of the microgels, and thus, the microgel particles reduce in size, which in turn influences their interfacial behavior [129]. Li et al. studied the adsorption kinetics of PNIPAM microgels at the oil/water interface and reported that the deformability of the microgels at the interface is an important factor for emulsion stabilization [130].

Increasing attention has been paid to developing new microgel-stabilized emulsions based on functional materials, including scaffolds for tissue engineering [131,132,133,134,135], nanoporous films [136], switchable catalyst system, and capsules with tunable permeability for controlled release applications [125,137]. Functional nanoparticles are utilized during the material development step to make the materials multi-functional and more suitable for the desired application [132,133]. In this section, we will present a brief overview of novel functional materials that have been developed by using microgel-stabilized emulsions.

### 4.1. Microgel-Stabilized Emulsions as Switchable Catalytic Systems

Microgels loaded with metal nanoparticles have gathered increasing attention in recent years for catalytic applications [20,138,139,140]. Pich et al. developed PVCL- and acetoacetoxyethyl methacrylate (AAEM)-based microgels, which were used as templates for controlled formation and site-specific deposition of gold nanoparticles and PEDOT nanorods/Au nanoparticles [20,141]. The developed systems showed excellent catalytic activity for the reduction of *p*-nitrophenol to *p*-aminophenol. Lu et al. fabricated polystyrene@PNIPAM microgels loaded with silver nanoparticles, and the catalytic activity of the system for *p*-nitrophenol conversion was modulated as a function of temperature [44]. At low reaction temperatures, the microgels were swollen and the reactants could reach the metal catalyst, leading to *p*-aminophenol formation. On the other hand, at higher reaction temperatures, PNIPAM chains were shrunken, which slowed down the diffusion of reactant, leading to the decrease of reaction rates. Biffis et al. developed vinylpyridine-functionalized microgels for Au nanocluster formation, and the developed hybrid particles were used for the aerobic oxidation of primary and secondary alcohols in water [142].

On demand stabilization and breakage of microgel-stabilized emulsions are of great industrial importance as these emulsions can be used as switchable catalytic systems [143]. For example, a water-based enzyme converts the oil-based substrate to the product at an accelerated reaction rate owing to the presence of a large interface. Afterwards, the emulsion can be easily broken by applying an external stimulus, and the product can be recovered while the microgels and enzymes can be reused for the next conversion cycle.

**Figure 5:**
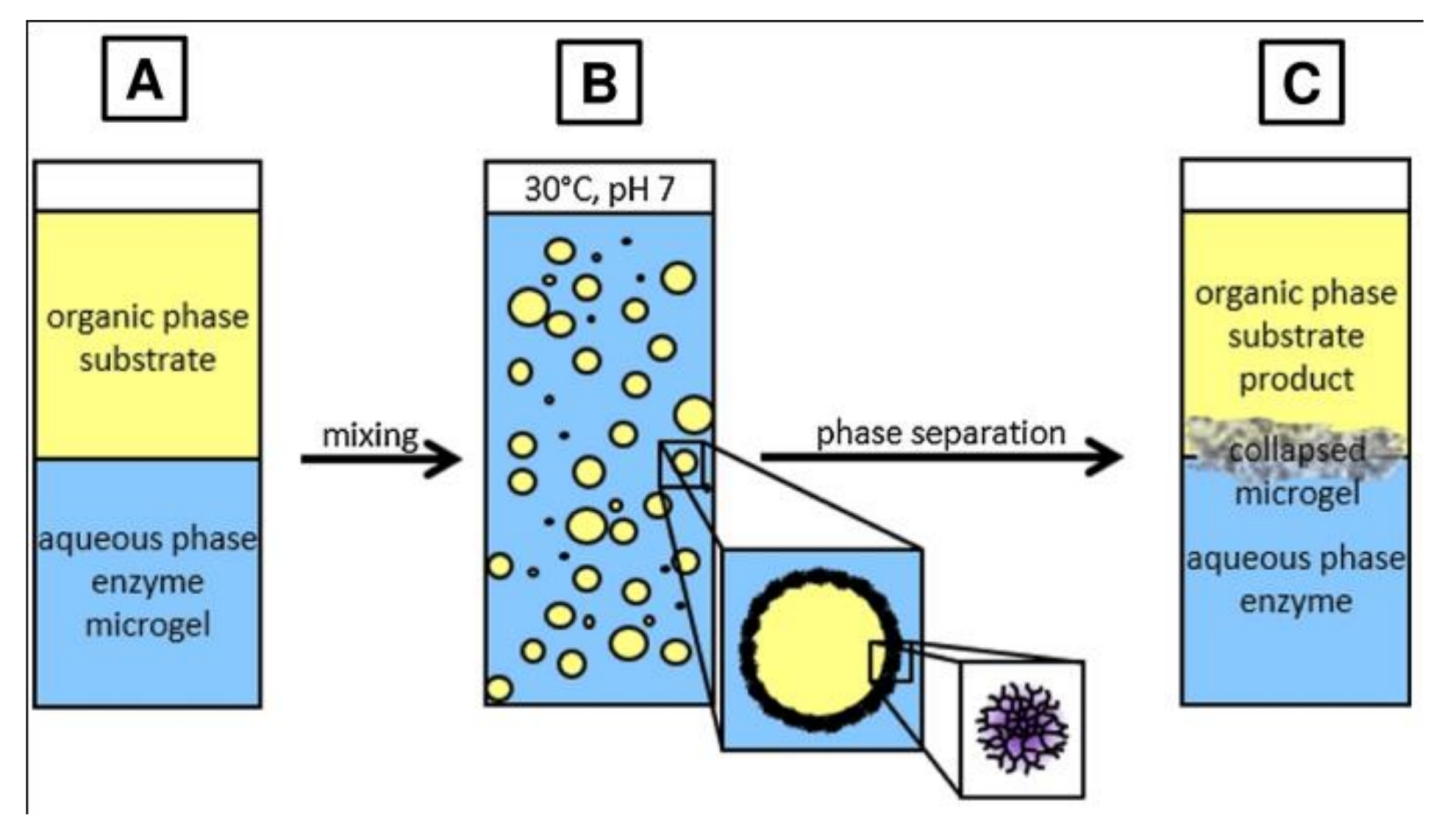
Two-phase system (**A**); the stabilization of the emulsion droplets with the responsive microgels and the enzyme reaction take place in the emulsion (**B**); breaking the emulsion by increasing the temperature (**C**). (Reprinted with permission from [144]).

Inherent enantioselectivity and mild reaction conditions are the prime characteristics of biocatalysis, which is used to produce enantiopure substances. However, many substrates of interest are poorly soluble in water, whereas enzymes typically prefer an aqueous environment. To achieve an efficient two-phase enzymatic reaction, it is necessary to improve the interface area by preparing an emulsion and preventing the enzyme denaturation at the interface. However, once again, at the end of the reaction the emulsion needs to be broken under moderate conditions to collect the products and recycle the enzymes present in aqueous phase. To address this aim, Wiese et al. formulated PNIPAM/PNIPMAM-based copolymer microgels present in the buffer triethanolamine hydrochloride (TEA·HCl) with enzymes to stabilize the oil phase containing the reaction substrate (Figure 5) [144]. Here, alcohol dehydrogenase from Lactobacillus brevis was used as the model enzyme for reducing acetophenone to (*R*)-phenylethanol, and the emulsion could be easily destabilized by increasing the temperature once the conversion was over [144,145].

**Figure 6:**
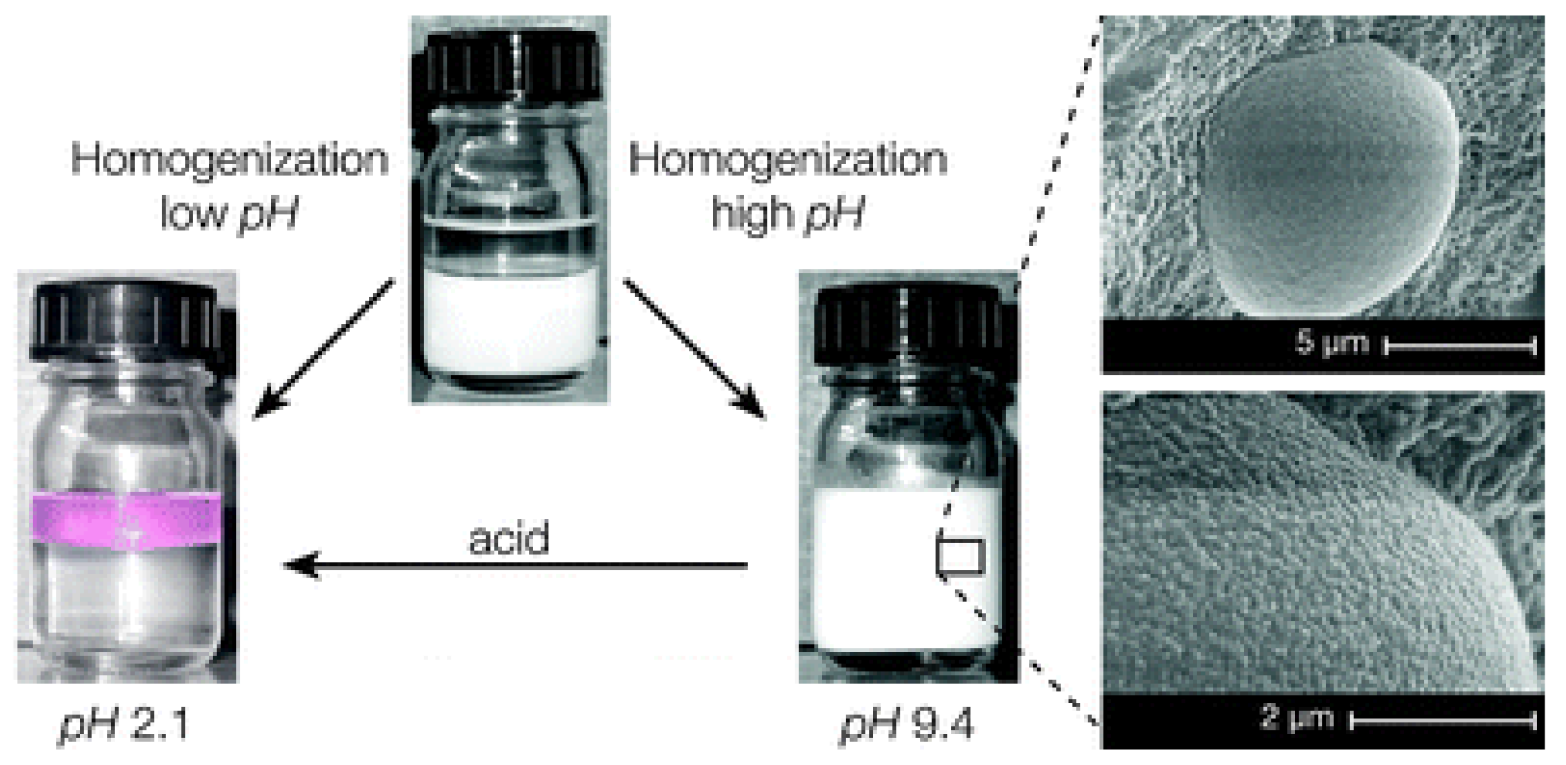
Effect of pH on the formation of octanol-in-water emulsions after 48 h at *T* = 25 °C. The oil phase in the leftmost photograph is strongly colored by the dye-labeled particulate stabilizers. The inset shows freeze–fractured SEM pictures of a particle-covered droplet in an emulsion prepared at pH 9.4. (Reprinted with permission from [124]).

Brugger et al. developed temperature- and magnetic-field-responsive PNIPAM-based microgels loaded with Fe_3_O_4_ nanoparticles, which stabilized the oil/water interface consisting of both polar and nonpolar oils [146]. These emulsions could be easily controlled remotely by applying high frequency magnetic fields leading to phase separation. Further, Ngai et al. designed PNIPAM/MAA microgels stabilized octanol in water emulsions, which can be remotely controlled by both pH and temperature stimuli (Figure 6) [124]. It was observed that stable emulsions are formed above pH 6, while interfacial activity decreases upon lowering the pH. The stable emulsions prepared at neutral pH show destabilization upon increasing the temperature to 60 °C, whereas at pH > 8 the emulsion is stable upon heating. The unique feature of controlling the emulsion stability by both temperature and pH makes the developed system a suitable candidate for on-demand, switchable catalytic systems.

### 4.2. Microgel-Stabilized Emulsions for Designing Adaptive Capsules

Using responsive materials that undergo reversible transitions upon stimulation provided by temperature, pH, light, magnetic field, ionic strength, etc. provides us with the possibility of designing capsules with controlled wall permeability, mechanical properties, degradation, and cargo release. As the microgels display high chemical functionality, ease of synthesis, possibilities of post-modification and incorporation of functional nanomaterials, and surface-active properties, they are perfect candidates to be used for developing capsules with desired properties. Recently, Lawrence et al. fabricated PNIPAM/AA microgel-based hollow capsules that can undergo expansion and contraction upon heating and cooling, respectively [147]. Microgels assemble at the octanol/water interface and are electrostatically interlinked by the use of diblock copolymer poly(butadiene-*b*-*N*-methyl 4-vinyl pyridinium iodide). When the temperature is increased up to 42 °C, the capsules exhibit reversible reduction in radius up to 13%. Irreversible reduction in radius (up to 40%) is observed when the temperature is increased up to 64 °C, owing to the increased van der Waals attraction among the microgel particles in their collapsed state. The developed capsules can be used as microscopic pumps or actuators [147].

**Figure 7:**
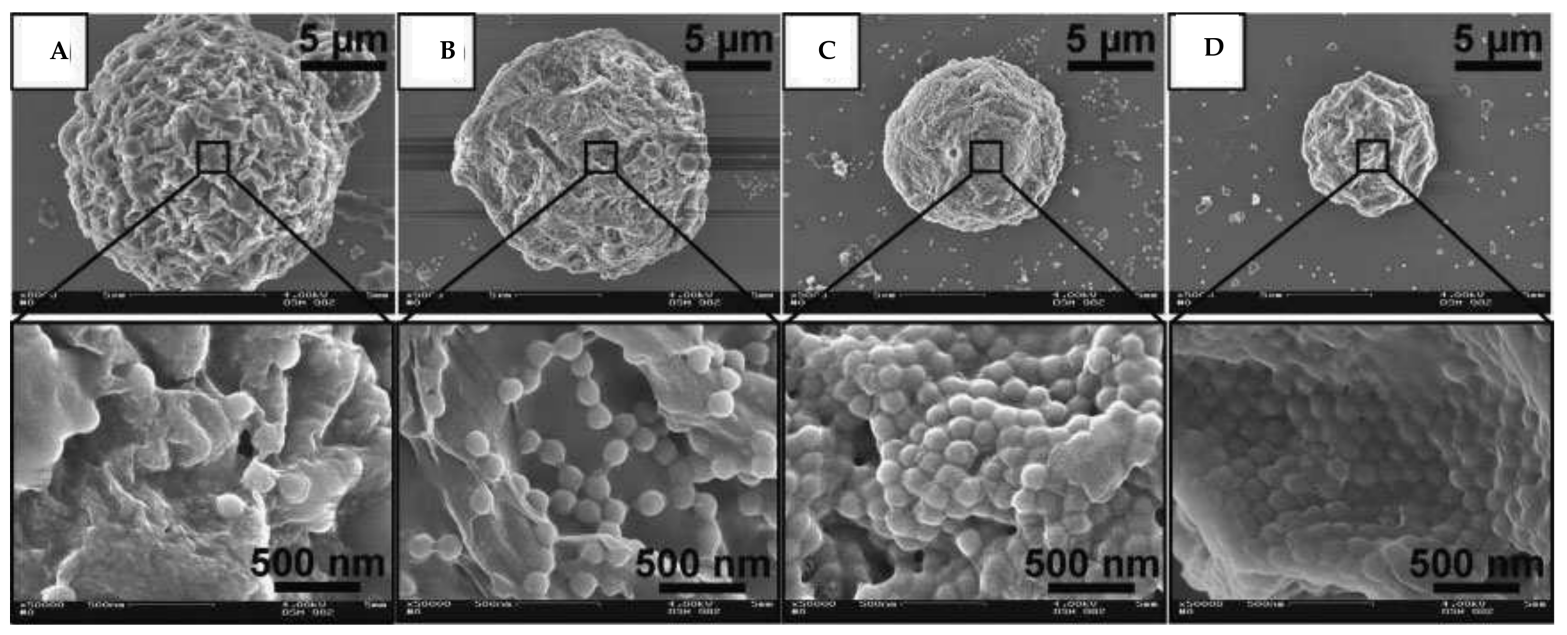
SEM images of capsules prepared by varying the microgel concentration in the aqueous phase (*C*_PHBV_ = 30 g/L): (**A**) 0.042 g/L; (**B**) 0.083 g/L; (**C**) 0.125 g/L; (**D**) 0.167 g/L (high resolution images illustrate the capsule wall morphology). (Reprinted with permission from [148]).

Berger et al. reported a facile route to develop temperature-responsive capsules based on PVCL microgels [148]. The microgel particles present in the aqueous phase stabilize the chloroform droplet containing a biodegradable polymer poly(4-hydroxybutyrate-*co*-4-hydroxyvalerate) (PHBV) and eventually become integrated into the capsule wall. Scanning electron microscopy images of various capsules prepared by using different microgel concentrations during the preparation reveal that the capsule size decreases with increasing microgel amounts due to their surface-active properties leading to smaller droplet formation (Figure 7) [148]. The high magnification images show that the capsule wall consists of microgels embedded in the PHBV layer, and compact microgel packing is observed with increasing microgel amounts in the aqueous phase. The VPTT of microgels is 32 °C, while the VPTT of microgel-based capsules is shifted to higher values due to the restricted motion of the microgel chains in the brittle PHBV layer. These capsules were loaded with FITC-Dextran, and its release was observed as a function of temperature. It was reported that the microgels were swollen at 25 °C, leading to fast release of the cargo, while at 70 °C the microgels shrink, and capsule wall permeability decreased resulting in slower FITC-Dextran release.

**Figure 8:**
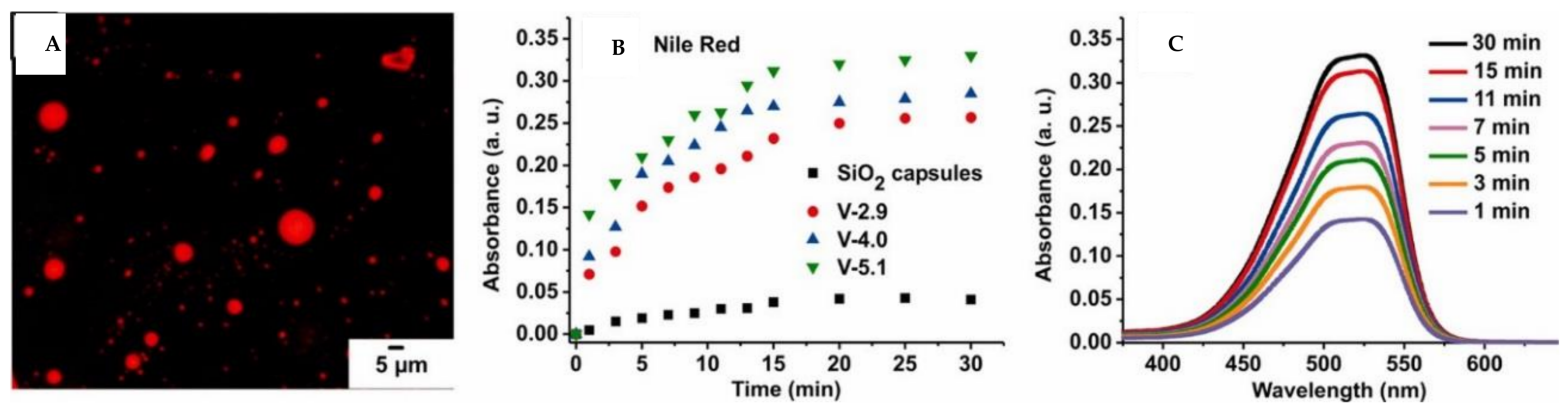
Fluorescence microscope image of Nile-red-loaded V-5.1 capsules (prepared by using 5.1 wt % microgels during the synthesis step) (**A**); The Nile red release profile given by absorbance as a function of time (**B**); UV–Vis spectra of the Nile red released from the V-5.1 capsules in toluene (**C**). (Reprinted with permission from [125]). V-5.1, V-4.0, and V-2.9 capsules were prepared by using 5.1 wt %, 4.0 wt %, and 2.9 wt % microgels, respectively, during the synthesis step.

**Figure 9:**
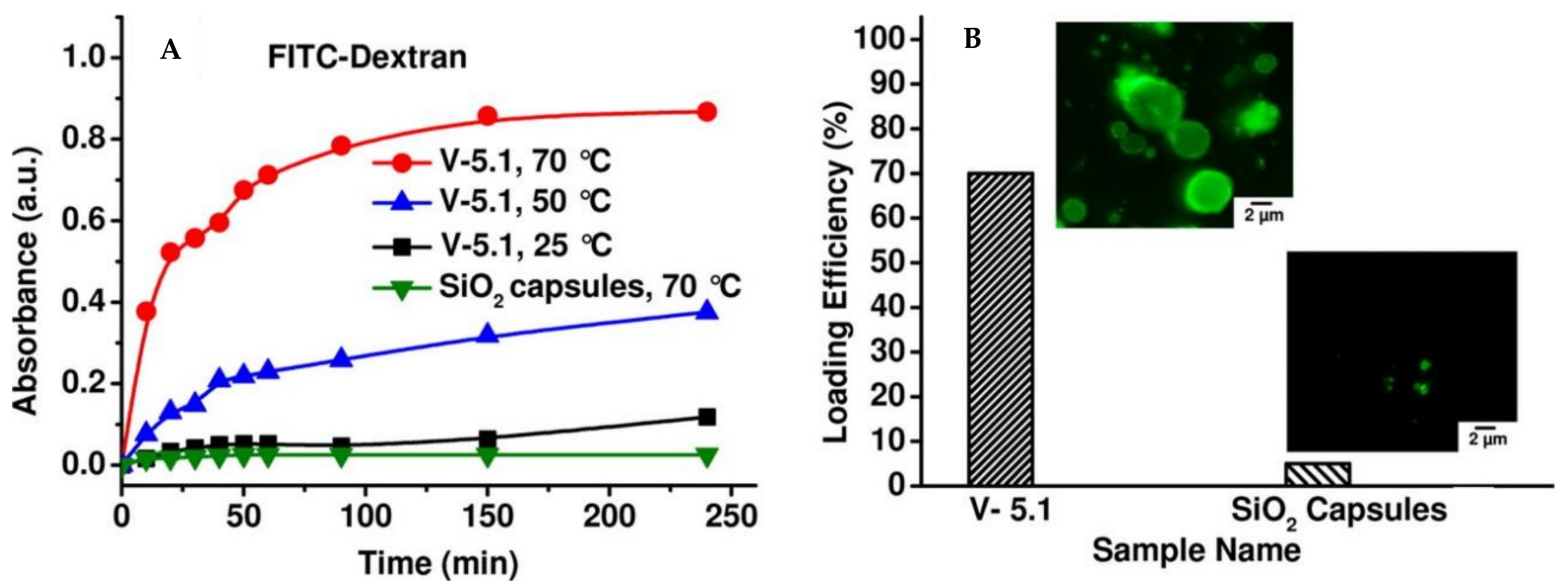
Release profiles of FITC-Dextran from the V-5.1 capsules (prepared by using 5.1 wt % microgels during the synthesis step) detected at different temperatures (**A**); loading efficiencies of the capsules (insets show fluorescence images of the capsules loaded with FITC-Dextran) (**B**). (Reprinted with permission from [125]).

Agrawal et al. developed microgel/silica adaptive hybrid capsules with controlled permeability [125]. Here, amine-functionalized PVCL microgel-stabilized polyethoxysiloxane (PEOS) containing toluene droplet and the subsequent hydrolysis/condensation of PEOS at the interface led to the composite capsules where microgels were embedded in the silica wall. The microgels were found to be temperature-sensitive even after their incorporation in the silica layer. However, their VPTT was shifted to a higher temperature due to the restricted chain dynamics. To investigate the capability of these capsules to operate both in organic and aqueous media, the release of the hydrophobic dye Nile red in toluene and hydrophilic FITC-Dextran in water was studied. Figure 8A presents Nile-red-loaded fluorescent capsules dispersed in water. The release of Nile red from the capsules into toluene medium was investigated by UV–Vis measurements, which showed an increase in absorbance characteristic of Nile red as a function of time (Figure 8B,C). In contrast, the pure silica capsules showed almost no release even after 30 min. Further, the release of FITC-Dextran in the aqueous medium was investigated as a function of temperature (Figure 9A). The experimental results clearly indicated that the release of FITC-Dextran was strongly temperature-dependent. Here, the faster release with the increasing temperature was probably due to the faster diffusion and shrinking of microgels providing bigger local channels. In contrast with pure silica capsules, the loading efficiency of microgel-based capsules is 70%, owing to easy diffusion of FITC-Dextran via microgel-based transport channels embedded in capsule wall (Figure 9B).

**Scheme 3:**
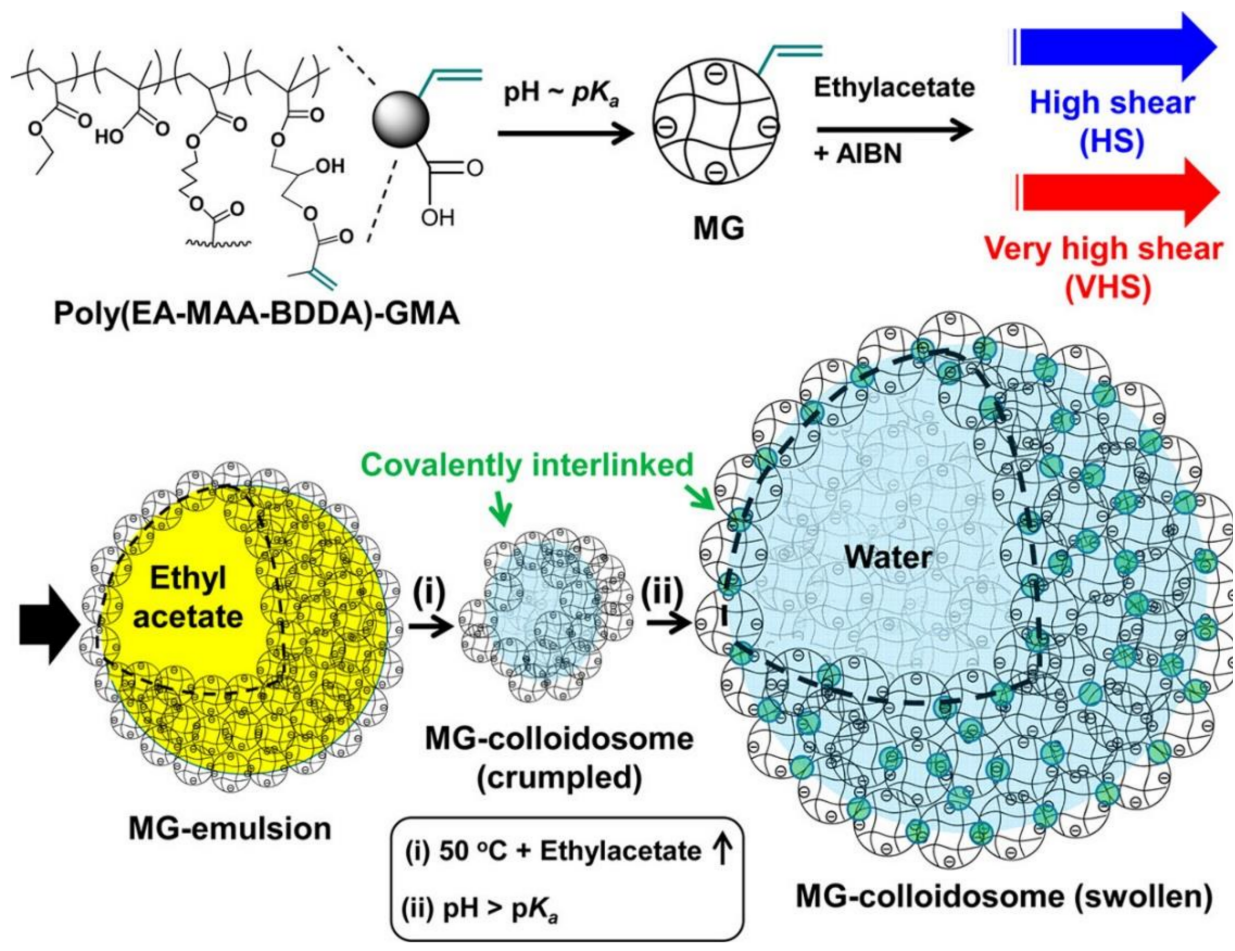
Preparation of pH-responsive capsules using (poly(EA-MAA-BDDA)-GMA) microgels. Microgel-stabilized ethyl acetate-in-water emulsions containing an AIBN initiator and the stabilized emulsion was heated to covalently interlink the microgels via free-radical coupling of surface vinyl groups. (Reprinted with permission from [149]).

Saunders et al. designed pH-responsive poly(ethyl acrylate-*co*-methacrylic acid-*co*-1,4-butanediol diacrylate)-glycidyl methacrylate (poly(EA-*co*-MAA-*co*-BDDA)-GMA) microgel-based capsules (Scheme 3) [149]. Here, microgels stabilize the interface and are interlinked by thermal free-radical coupling caused by azoisobutyronitrile (AIBN) initiator present in the oil phase. When the pH was increased above 6, the capsules displayed swelling and robustness by avoiding the rupture. A range of FITC-dextran polymers was used to investigate the size-dependent permeation through the capsule wall. The estimated pore diameter of the capsules was between 6.6 nm and 9.0 nm at pH 6.2. As the microgels contained plenty of carboxylic groups, they can be post-modified to obtain the desired functionalities, and these capsules can be used for cosmetics, photonics, and delivery applications. Further, Saunders et al. used poly(ethylacrylate-*co*-methacrylic acid-*co*-1,4-butanediol diacrylate)-glycidyl methacrylate (poly(EA-*co*-MAA-*co*-1,4-BDDA)-GMA- and poly(methyl methacrylate-*co*-methacrylic acid-*co*-ethyleneglycol dimethacrylate)-GMA (poly(MMA-*co*-MAA-*co*-EGDMA)-GMA)-based double crosslinked microgels to design pH-responsive capsules [150]. These capsules were biocompatible and were synthesized at pH 6.8. Therefore, these capsules can be used for the encapsulation and pH-based release of biological substances. De Laporte et al. fabricated degradable capsules based on microgels prepared by photo-crosslinking six-armed acrylated star-shaped poly(ethylene oxide-*stat*-propylene oxide) pre-polymer using the microfluidic technique [151]. These capsules were used to directly encapsulate the large amounts of biological molecules and drugs.

## 5. Functional Microgel-Based Antifouling Coatings

The adsorption of biomolecules, cells, and microorganisms on various substrates, also known as “fouling”, has emerged as a critical problem in recent years [152,153,154]. Hence, increasing attention has been paid to antifouling substances as they can resist the aforementioned adsorption, which is important for various areas, including biomedical implants, drug delivery, biosensors, bioseparation, and marine coatings [155,156,157,158].

A major concern for using nanomaterials, including microgels as drug delivery carriers is their short-term stability in blood circulation and recognition by the immune system because of nonspecific protein adsorption [159,160]. In order to address this problem, various microgels consisting of antifouling polymers such as PEG [161], tetraglyme [162,163], dextran [164,165], glycerol Dendron [166], mannitol [167], and poly(*N*-(3-sulfopropyl)-*N*-(methacryloxyethyl)-*N*,*N*-dimethylammonium betaine) (polySBMA) [168] have been fabricated. These antifouling substances reduce the adsorption of proteins from blood on the carrier surface and thus delay their recognition by the immune system [169]. This helps the microgels to efficiently reach the target site and cross the cell membrane for controlled release of drug. Jiang et al. developed zwitterionic poly(carboxybetaine methacrylate) (pCBMA)-based microgels via the inverse microemulsion free-radical polymerization method [170]. These microgels exhibited excellent stability in 100% fetal bovine serum for up to 18 h and did not induce cell toxicity upon 4 h of incubation in phenol red-free medium. Zheng et al. designed poly(*N*-(2-Hydroxyethyl) acrylamide) (HEAA)/acrylic acid (AA)-based microgels, which showed potential utility as in vivo drug delivery [171]. The experimental results showed that polyHEAA-based microgels showed resistance to protein adsorption while maintaining their stability in 100% human blood plasma for up to 30 days in vitro.

Medical device integration and performance is highly influenced by inflammatory host responses caused by protein adsorption, leukocyte activation, cytokine release, and fibrous encapsulation of the implant. Bridges et al. developed PNIPAM/AA microgel-based coatings on poly(ethylene terephthalate) (PET) used as a model implant substrate [172]. These coatings significantly reduced in vitro fibrinogen adsorption, primary human monocyte/macrophage adhesion and spreading, leukocyte adhesion, and expression of pro-inflammatory cytokines (TNF-α, IL-1β, MCP-1). The coated and uncoated substrates were implanted subcutaneously in rats for four weeks. The analysis of explants indicated that the microgel film reduced the chronic inflammation compared to unmodified PET substrates, thus showing their potential for attenuating adverse host responses [173]. Further, polyethylene glycol-containing polyurethane hydrogel coatings have also been formulated for neural electrodes [174].

**Figure 10:**
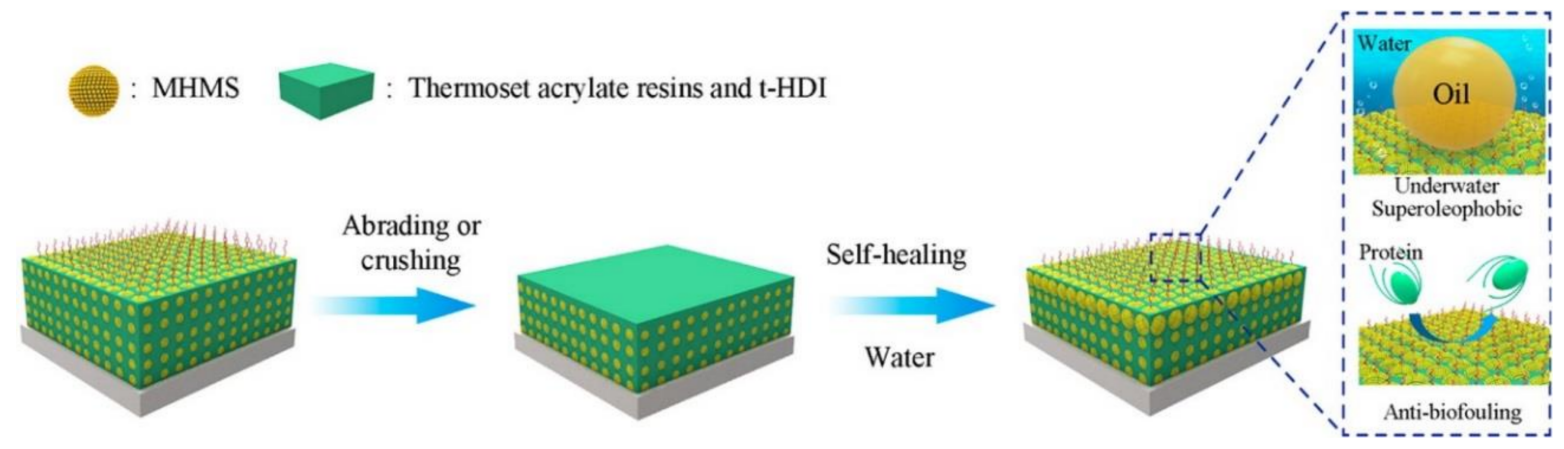
Schematic representation of the working principle of the self-repairing underwater superoleophobic/antifouling coating. (Reprinted with permission from [175]).

In order to address the problem of marine biofouling on shipping and leisure vessels, heat exchangers, oceanographic sensors, and aquaculture systems, Chen et al. developed PNIPAM/MAA/poly(ethylene glycol) diacrylate (PEGDA)-based microgels for coatings [175]. The modified hierarchical microgel spheres (MHMS) are crosslinked in acrylate matrix using 1,6-hexamethylene diisocyanate trimer (t-HDI), leading to self-healing, oil repellent, antifouling coating (Figure 10) [175]. These coatings not only show prominent underwater superoleophobicity but also exhibit exceptional antifouling property owing to its 3D-ordered structure obtained by the self-assembly of the microgels. These microgels contain hydrophilic copolymeric chains grafted on the surface, which also play a crucial role for antifouling properties. The ability of the developed material to regain the oil- and biofouling-resistant properties once its surface is mechanically damaged opens a new pathway for self-healing antifouling coatings.

## 6. Assembly of Cell-Laden Microgels for Tissue Engineering Applications

In recent years, microgels and microgel-based composites have emerged as the candidate of special interest for tissue regeneration owing to their diverse chemical functionality, ease of synthesis and handling, biocompatibility, and cost effectiveness [176,177]. Furthermore, the numerous possibilities of incorporating small active groups, functional nanoparticles, growth factors, and biological active substances increase their versatility for tissue engineering application. Hence, an increasing attention has been paid to develop microgel-based systems with improved cell adhesion and cell growth [178,179,180,181]. The concept of bottom up tissue engineering has been applied to develop complex 3D architecture based on the assembly of cell-laden microgels, thus providing the tissue like complexity [182,183].

Khademhosseini et al. fabricated photo-crosslinked graphene oxide (GO)/gelatin methacrylate (GelMA) hybrid microgels based on the microfluidics approach, and these microgel particles were loaded with NIH-3T3 fibroblasts cells [184]. It was observed that the use of grapheme oxide allowed the development of complex artificial tissues with controlled mechanical and electrical properties. Cell adhesion, spreading, and proliferation was also promoted, thus indicating the potential of the developed material for designing more complex constructs, such as blood vessels, skin, skeletal muscle, and connective tissue. Doyle et al. reported the formation of PEG diacrylate-based anisotropic microgels loaded with NIH-3T3 fibroblast cells by the stop flow lithography technique (SFL) [185]. The SFL method is advantageous as compared with the continuous flow lithography technique as it does not require short polymerization time, slow flow rate, or highly concentrated prepolymer solution, and hence, the toxicity to the cells is reduced. These anisotropic microgels can be further self-assembled based on the differences in surface energy at different areas, thus generating complex tissue architectures.

**Figure 11:**
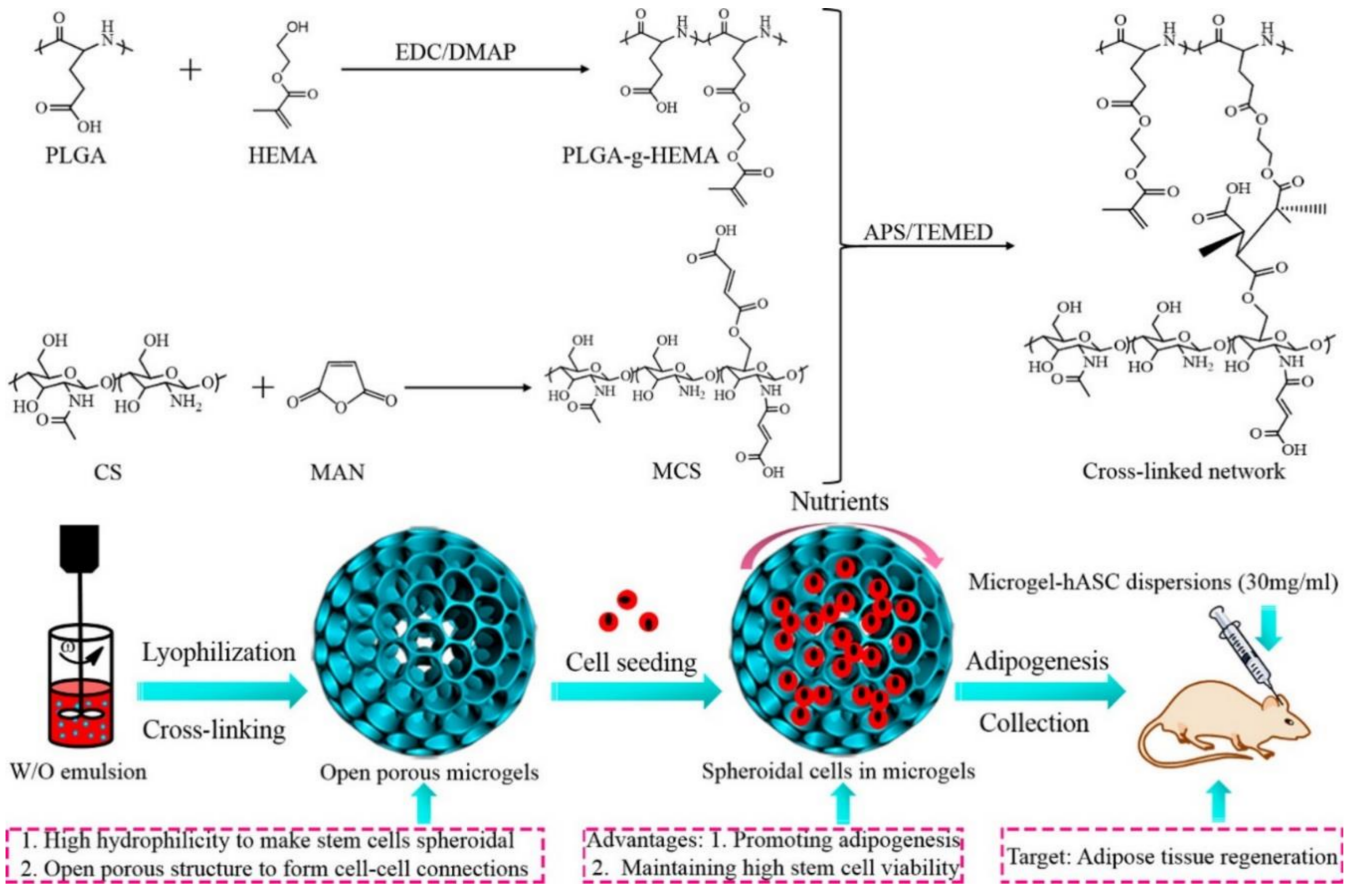
Schematic illustration of the preparation of PLGA-*g*-HEMA, MCS, and open porous microgels. After cell seeding, spheroidal cells were maintained in the open porous microgels. Human adipose stem cells dispersions were injected to evaluate the formation of adipose tissue after 14 days of adipogenic induction in vitro. (Reprinted with permission from [188]).

Weitz et al. synthesized multicompartment microgels loaded with different cells using the microfluidic approach [186]. The loading of microgels with stem cells adjacent to niche cells in other compartments provides the potential to understand the intercellular communication, which is useful for cell biology, stem cell therapy, and tissue engineering. Further, Utech et al. designed monodisperse alginate microgels of 10–50 µm in diameter and crosslinked with calcium ions via the droplet microfluidics method [77]. Living mesenchymal stem cells were encapsulated in these microgels, which showed stable growth and proliferation. The mechanical properties of these microgels can be tuned by adjusting the crosslinker amount and the nature of the alginate chains. These small microenvironments can be further self-assembled to develop more complex biological structures. Ma et al. presented the synthesis of furylamine- and tyramine-grafted hyaluronic acid molecule-based microgels via microfluidics-assisted enzymatic crosslinking and/or Diels–Alder click chemistry [187]. Enzymatic crosslinking and click chemistry crosslinking allow higher chemical selectivity and milder reaction conditions along with tunable gelation time and elasticity. ATDC-5 cells were used as model cells inside microgels, and the cells showed high viability for up to 14 days after the culture. For the first time, Xia et al. reported the fabrication of poly(l-glutamic acid)-*g*-2-hydroxyethyl methacrylate (PLGA-*g*-HEMA)- and maleic anhydride-modified chitosan (MCS)-based microgels for adipose tissue engineering [188]. PLGA-*g*-HEMA was synthesized through esterification reaction between PLGA and HEMA, while MCS was synthesized in a one-step reaction between maleic anhydride and chitosan (Figure 11) [188]. Here, adipogenic differentiation and adipose tissue regeneration were promoted by the formation of extensive intercellular interactions within the microgels. The experimental results showed that the microgels could maintain high cell viability up to 14 days, and adipose tissue was regenerated after 12 weeks of implantation.

Duschl et al. developed artificial surfaces with easy to process, robust, patterned thermoresponsive microgel coating for controlled adhesion and cell growth [189]. PNIPAM-based microgels synthesized by microfluidic technique were used for the fabrication of patterned coating employing automated nanodispensing and microcontact printing. Degree of swelling, elastic modulus, and adhesion properties were investigated below and above the VPTT of the microgels. The grown cells could be detached by applying the temperature stimulus, and it could be used to perform noninvasive cell experiments [107]. Experimental results indicated that the cells were well-spread on the surface at 37 °C, while they were completely detached at 25 °C due to high water content, increased repulsive interactions, and unfavorable modulus.

**Figure 12:**
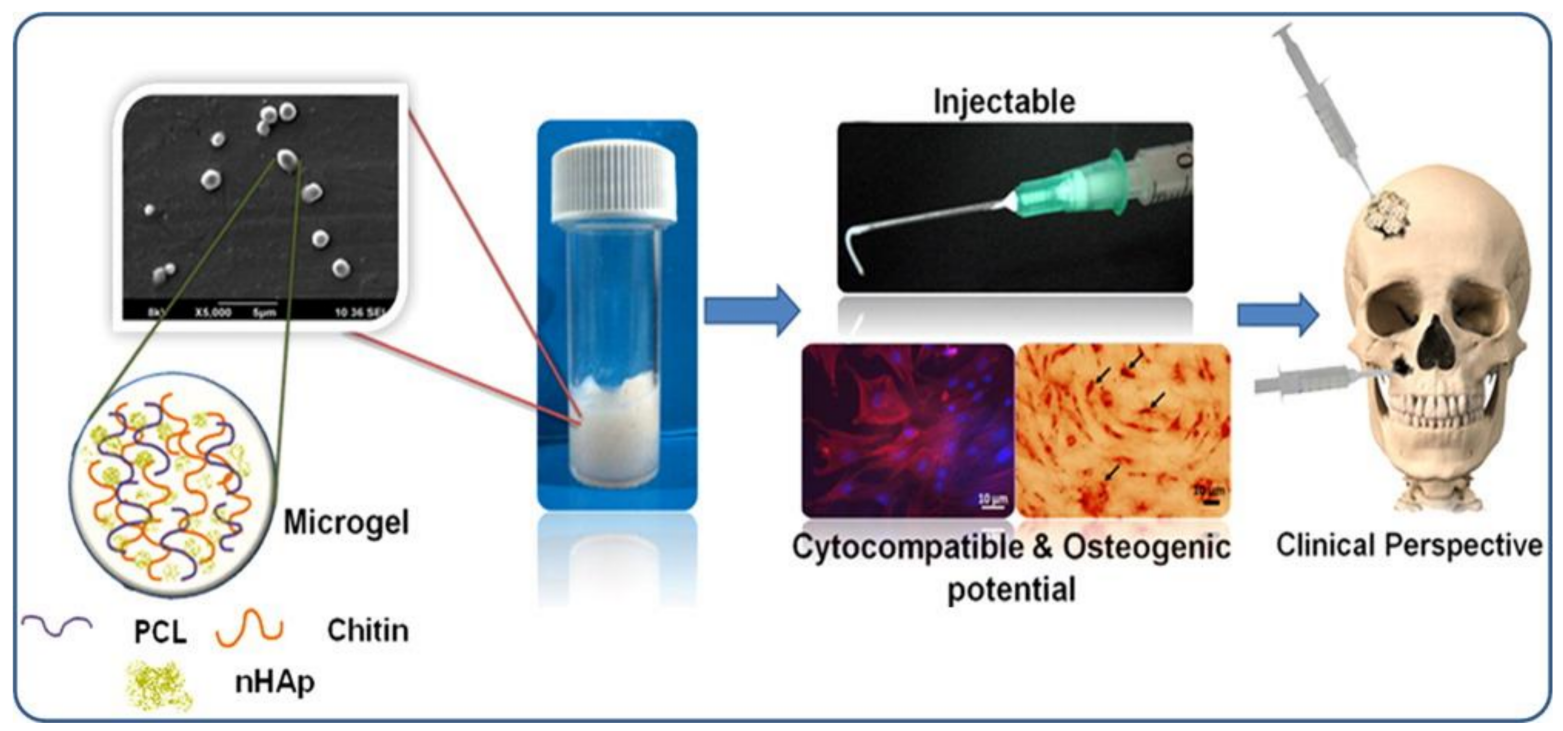
Injectable chitin-PCL-nHAp microgels showing cyto-compatibility along with osteogenic potential for bone tissue engineering. (Reprinted with permission from [190]).

Jayakumar et al. developed chitin-poly(ε-caprolactone) (PCL)/nanohydroxyapatite (nHAp) composite-based injectable composite microgels, which showed improved elastic modulus and shear thinning behavior [190]. These microgels exhibited enhanced cell migration and proliferation along with increased mineralization and thus proved to be a potential candidate as injectable material for regeneration of deeper and more complex bone defects (Figure 12).

A straightforward magnetic field assisted technology has been developed to assemble the microgels to construct complex biological 3D architectures [191]. Xu et al. designed magnetic nanoparticles and NIH-3T3-cells-loaded gelatin methacrylate-based microgels, which were successfully assembled into multilayer 3D constructs with controlled geometry and number of layers by simply using external magnetic fields [192]. Tasoglu et al. developed heterogeneous complex structures by self-assembly of magnetoceptive gels using paramagnetism of free radicals as a driving and thus opened a new pathway for addressing bottom-up tissue engineering [193].

## 7. Conclusions and Future Perspectives

Owing to their stimuli sensitive properties, ease of synthesis, possibilities of post-modification, compartmentalization, chemical functionality, softness, and deformability, microgels have been extensively used for designing functional materials. In this review we highlighted various approaches for fabricating microgels with desired properties, which exhibit interesting interfacial activity. These responsive microgels can deform at the oil/water interface and can lead to stabilized emulsions, which can be further used as switchable catalyst systems and adaptive capsules for controlled release applications.

To envision the successful application of this particular class of colloids in designing new products for biomedical applications, interdisciplinary research needs to be carried out by combining the approaches from various disciplines, such as organic and polymer chemistry, material science, and biomedical engineering, etc. Designing microgels and microgel-based composites loaded with drugs, growth factors, and biologically active substances while maintaining the ease of synthesis and cost effectiveness makes them an indispensable platform for biomedical applications.
